# Empathy Modulates the Rewarding Effect of Mimicry

**DOI:** 10.1038/srep27751

**Published:** 2016-06-14

**Authors:** J. Neufeld, B. Chakrabarti

**Affiliations:** 1 Centre for Integrative Neuroscience and Neurodynamics, School of Psychology and Clinical Language Sciences, University of Reading, Reading, United Kingdom; 2 Center of Neurodevelopmental Disorders, Karolinska Institutet (KIND), Stockholm, Sweden

## Abstract

We tend to like those who mimic us. In this study we formally test if mimicry changes the reward value of the mimicker, using gaze bias as a proxy for reward. Previous research has demonstrated that people show gaze bias towards more rewarding targets, suggesting that gaze bias can be considered a proxy for relative reward value. Forty adults participated in a conditioning task, where they were mimicked by one face and ‘anti-mimicked’ by another. Subsequently, they were found to show gaze-bias towards faces that mimicked them compared to those that did not, in a preferential looking task. The strength of this effect correlated positively with individual levels of trait empathy. In a separate, similar task, these participants showed a gaze bias for faces paired with high vs low monetary rewards, thus validating the use of gaze bias as a proxy for learnt reward. Together, these results demonstrate that mimicry changes the reward value of social stimuli, and empathy influences the extent of this change. This can potentially inform conditions marked by deficits in forming social bonds, such as Autism.

## Introduction

Mimicry has been suggested to function as a “social glue”, a key mechanism that helps to build social rapport^1^,^2^. It leads to increased feeling of closeness toward the mimicker^3^,^4^ as well as greater liking and increased prosocial behaviour^5^,^6^,^7^,^8^, suggesting that being mimicked is inherently rewarding. Mimickers are perceived as more persuasive^9^ compared to non-mimickers and are trusted more^10^,^11^. Being mimicked not only changes people’s attitude towards the mimicker, but also increases their perceived closeness to others in general^12^ and makes them more assimilative^13^. In sum, mimicry helps social bonding and smoother social interaction, making it beneficial for both the mimicker and mimickee^3^. The rewarding nature of mimicry is further supported by a study showing increased activation and functional connectivity of brain areas involved in reward processing in adults when mimicked compared to not being mimicked^14^. The link between mimicry and reward seems to exist already early in life: babies look and smile longer at adults who are imitating them compared to adults imitating another baby or performing only temporally but not structurally congruent movements^15^. Parents routinely exhibit imitative behaviour with their babies, in order to entertain them and attract their attention. It has been suggested that imitation serves as a fundamental mechanism for understanding others’ actions and intentions and is therefore essential for the development of empathy^16^. Understanding the reward response to imitative behaviour can therefore be informative for conditions marked by deficits in empathy, such as Autism Spectrum Conditions (ASC). It is worth noting however, that the benefits of mimicry on social cognition is context dependent. In some contexts, intentional mimicry can impair emotion recognition^17^ or distinction of true from faked emotions^18^, while in others, being able to spontaneously mimic can enhance emotion recognition^19^, especially in women^20^.

Importantly, the link between reward and mimicry is bidirectional, i.e. we also mimic people more if we like them more^21^,^22^,^23^. Direct support for this view comes from a study showing that increasing the reward value of a face facilitates spontaneous facial mimicry in adults: faces associated with higher reward (winning) were mimicked more compared to the faces associated with lower reward (losing)^24^. Using the same conditioning paradigm in an fMRI study, Sims *et al*. reported greater functional connectivity between reward- and mimicry related brain areas (identified through independent meta-analyses) when seeing smiling faces associated with high compared to low reward value. The same experimental paradigm when tested using electroencephalography (EEG) revealed greater mu suppression (suggested to be an index of cortical motor simulation) in response to faces conditioned with higher vs lower reward^25^. While the studies discussed above have tested the impact of systematically varying the reward value of social stimuli on spontaneous/automatic mimicry, the link has not been tested systematically in the other direction. That is, the extent of mimicry has not been systematically manipulated in order to test its impact on the reward value of social targets. Here we address this gap in the literature, by investigating the effect of being mimicked on reward value (measured using gaze bias in a preferential looking task). Longer gaze towards visual stimuli when presented side by side has been shown to be related to relative preference and positive evaluation^26^,^27^,^28^,^29^. In contrast to rating, it is less explicit and therefore less likely to be affected by reporting bias and similar psychological factors. Additionally, we test if trait empathy modulates this gaze bias. Trait empathy provides an index of individual differences in how well people understand and relate to others^30^. Previous studies have shown that individuals with higher trait empathy imitate more^4^,^31^ and that affective empathy modulates the effect of mimicking on prosocial behaviour^32^. In this study, we move beyond this known role of empathy in modulating the tendency to mimic, and hypothesise that the response to being mimicked is also modulated by trait empathy, i.e. mimicry is more rewarding to those people who are more sensitive to others’ facial expressions of emotion. If people low in trait empathy show a relatively weaker link between mimicry and reward, this might have implications for understanding conditions marked by deficits in empathy, such as ASC. Finally, to verify whether gaze bias (as used in our study) reflects learnt reward value, we run a separate experiment (Experiment 2) to test if classical reward conditioning with monetary reward influences gaze bias in a similar way.

## Aims and hypotheses

The overarching goal of this study is to test the mimicry-reward link and its relation to trait empathy. This goal is addressed through three aims: (1) To test whether mimicry conditioning increases gaze bias for mimicking vs anti-mimicking faces. (2) To investigate whether this gaze bias due to mimicry conditioning is modulated by trait empathy. It is hypothesised that individuals higher in trait empathy will have a greater relative reward value for mimicry, and (3) In a separate control experiment on the same sample of individuals, to confirm the validity of gaze bias as a metric for learnt reward value by testing whether reward conditioning (using monetary rewards) increases gaze bias for faces conditioned with high vs low rewards.

## Results

### Experiment 1: BeMim

#### Participant compliance

Analysis of the facial EMG data showed that all participants performed the correct facial expressions within the correct timeframe (i.e. after the instruction and before the beginning of the video stimulus) in more than 80% of trials during the conditioning.

#### Eye tracking results

**Figure 1:**
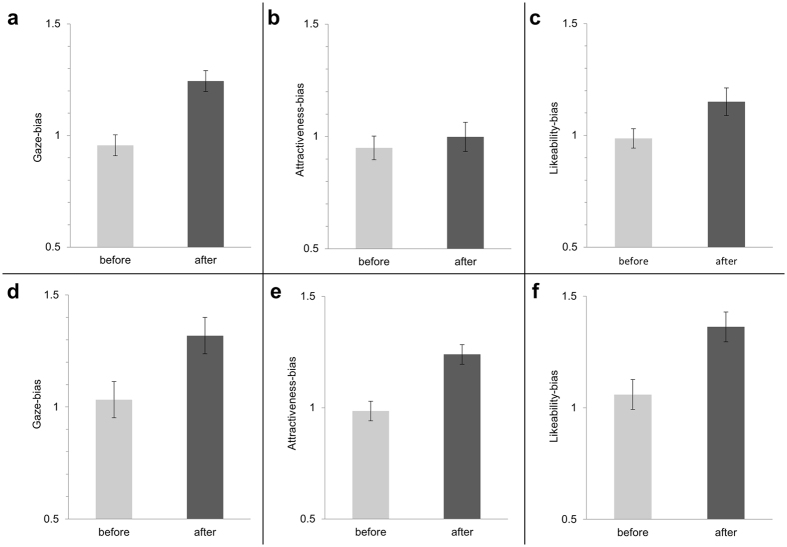
Group mean data of the three dependent variables: (**a**) gaze bias, (**b**) attractiveness bias and (**c**) likeability bias before and after BeMim conditioning and (**d**–**f**) before and after CARD conditioning. Error bars = within subject SEM.

**Figure 2:**
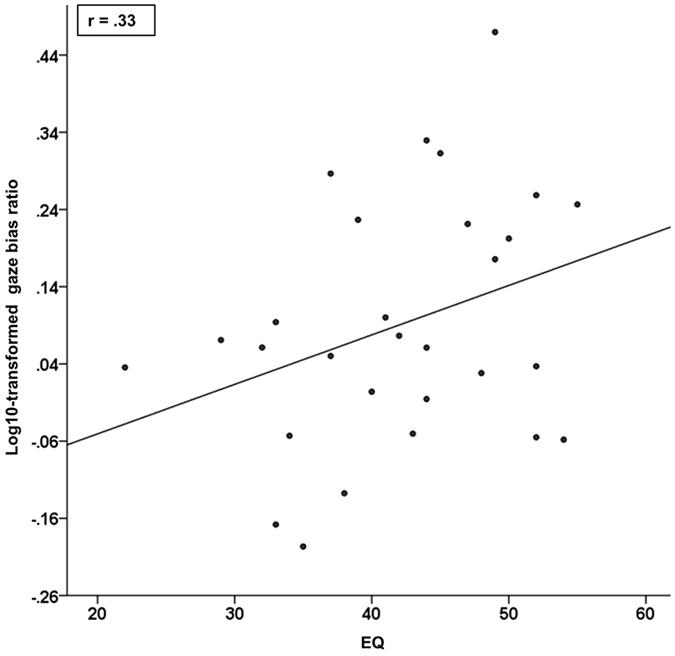
EQ correlation. Gaze bias for BeMim correlated positively with trait empathy (EQ), indicating that individuals with higher trait empathy showed greater preferential gaze to the mimicking face compared to the anti-mimicking face, after conditioning.

Gaze-bias for mimicking vs anti-mimicking faces was significantly greater (Wilcoxon Signed Rank test: z(37) = 2.889, p = 0.002) after conditioning (mean = 1.24) compared to before conditioning (mean = 96, see Fig. 1). Comparing the size of this conditioning effect using the size of a different reward conditioning on gaze bias as a prior^33^ revealed a Bayes factor of 38.33, indicating strong evidence for a conditioning effect (Bayes factor calculator: www.lifesci.sussex.ac.uk/home/Zoltan_Dienes/inference/bayes_factor.swf). Gaze-bias-ratio correlated positively with EQ (Pearson: r(28) = 33, p = 0.04; see Fig. 2).

#### Rating results

Attractiveness-bias was not significantly different (Wilcoxon Signed Rank test: z(44) = 1.027, p = 0.153) after conditioning (mean = 1.08) compared to before (mean = 1.05), nor was likeability-bias (before: mean = 1.01; after: mean = 1.16; z(44) = 1.420, p = 0.078. Attractiveness-bias ratio did not correlate significantly with EQ (Spearman’s Rho: r(33) = −0.055, p = 0.376), nor did log10-transformed likeability-bias-ratio (Pearson: r(33) = −0.104, p = 0.276).

### Experiment 2: CARD

#### Eye tracking results

Gaze-bias for high vs low reward-associated faces was significantly greater (Wilcoxon Signed Rank test: z(39) = 2.634, p = 0.004 after conditioning (mean = 1.28) compared to before conditioning (mean = 1.04). Comparing this conditioning effect to the same prior used in the BeMim experiment revealed a Bayes factor of 3.01, supporting the presence of a conditioning effect. Gaze-bias-ratio did not correlate significantly with EQ (Pearson: r(30) = 0.162, p = 0.188). Gaze-bias ratio showed no significant group difference between individuals who reported to have detected the conditioning pattern (winning with one face and losing with another) and those who did not (Mann-Whitney-U-Test: z(39) = 1.087, p = 0.139).

#### Rating results

Attractiveness-bias was significantly greater (Wilcoxon Signed Rank test: z(45) = 2.552 p = 0.011) after conditioning (mean = 1.21) compared to before (mean = 0.99), as was likeability-bias (before: mean = 1.06; after: mean = 1.33; Wilcoxon Signed Rank test z(45) = 1.713, p = 0.046). However, neither Attractiveness-bias-ratio nor Likeability-bias-ratio correlated significantly with EQ (Spearman’s rho: r(34) = −0.164, p = 0.170, and r(35) = 0.015, p = 0.465, respectively). Additionally, neither likeability-bias-ratio (Mann-Whitney-U-Test: z(45) = 0.465, p = 0.321) nor attractiveness-bias-ratio (Mann-Whitney-U-Test: z(45) = 0.822, p = 0.206) showed a significant group difference between individuals who reported to have detected the conditioning pattern and those who did not.

#### Validity check

Gaze-bias-ratio and likeability-bias-ratio were positively correlated (Spearman’s Rho: r(39) = 0.269, p = 0.046).

## Discussion

In this study, we systematically tested how manipulating the extent of mimicry associated with a face changes its reward value, measured using gaze bias and self-report ratings of likeability. In line with our hypothesis, we found that mimicry conditioning alters gaze bias within a preferential looking paradigm, in a way that the face associated with greater mimicry is preferred over the one associated with less mimicry (i.e. more ‘antimimicry’, in this case). As gaze bias has been shown to be related to preference and positive evaluation^26^,^27^,^28^,^29^, this metric is interpreted as a proxy measure related to the consummatory aspect of reward processing. To further validate the use of gaze bias as a proxy for learnt reward value in this context, we ran a second experiment with the same participants where high and low monetary rewards were associated with different faces. As expected, this paradigm showed that faces associated with high monetary rewards were associated with greater gaze-bias toward them in a preferential looking paradigm. The key findings of the study are discussed in detail below, in light of the extant literature.

### Mimicry modulates reward value of social targets

Greater gaze-bias towards the mimicking compared to the anti-mimicking face in experiment 1 indicates that repeatedly being mimicked effectively acts as a reward signal associated with the interaction partner. An effect of mimicry on gaze has been hypothesised by Kulesza *et al*.^17^. Further, the results are in line with former behavioural and neuroimaging studies showing that being mimicked can lead to increased feeling of closeness, prosocial behaviour and linking^3^,^5^,^12^ and increased activation in brain regions involved in processing rewards^14^,^34^,^35^,^36^. It also helps to explain why mimicking others has positive consequences for the mimicker such as being seen as more empathic and receiving higher tips^37^,^38^. All of these lines of evidence suggest that mimicry is a powerful tool for creating rapport. The current study establishes the rewarding effect of mimicry systematically within a standardized lab experiment by using a more implicit behavioural measure (in contrast to explicit rating) and while controlling for baseline biases by comparing this measure before and after conditioning. It has been shown that the mimicry-reward link is already present very early in life and is therefore thought to form an important prerequisite for typical social cognition^15^. Our results contribute to the evidence that this mechanism continues to play a role in social cognition in adults. This evidence thus provides a direct measure of the mimicry to reward link. The evidence for the link in the opposite direction, i.e. the impact of reward learning on mimicry has already been demonstrated in a set of previous studies showing greater mimicry and associated neural signals for faces associated with higher reward^24^,^25^,^39^. Together with these studies, the current results provide evidence for a bidirectional link between mimicry and reward system as a key mechanism of social cognition.

### Empathy modulates the strength of the mimicry–reward link

We found a positive correlation between EQ and gaze bias ratio as a result of mimicry conditioning. One interpretation of this finding is that for individuals high in empathy social stimuli, such as human faces, might be more salient which would increase the effectiveness of the conditioning. This interpretation is in line with finding of a former study, showing that the modulating effect of reward value on the imitation of hand movements correlated positively with EQ for human but not for robot hands^40^. This observation was interpreted to be due to the potentially higher saliency of human hands compared the robot hands in individuals with higher trait empathy. It is also possible that certain components of empathy, such as perspective taking, are required to decode ‘being mimicked’ as a positive social signal and therefore trait empathy modulates the conditioning effect by indexing decoding abilities rather than attention. However, both explanations are not mutually exclusive.

Further, it has been suggested that deficits in the linkage between mimicry and reward systems from early childhood can result in an impaired social cognition, leading to a deficient development of empathy^16^,^39^. Therefore individuals with a weakened link between mimicry and reward might not have developed the ability to empathize to the same extend as those with an intact linkage and therefore score low in EQ and show a decreased sensitivity to the conditioning. It is therefore possible that low EQ and decreased sensitivity to mimicry conditioning are driven by either (i) a third, more fundamental deficit in social cognition, rather than determining each other, or (ii) a weakened mimicry-reward link leads to deficits in empathy which later on lead to deficits in social cognition. Future studies are needed to test these two competing explanations. The role of empathy in social cognition is of special interest on the context of psychiatric disorders marked by deficits in empathy, such as ASC. Those with ASC might be less motivated to attend to social stimuli because they do not experience them as rewarding^41^, suggesting a cascade from a lack of attention to social stimuli to abnormalities in imitation and deficits in social cognition^42^,^43^. Further, individuals with ASC have been shown to be less emotionally affected by imitating others, suggesting an altered expression-emotion link^44^. Interestingly, there was no significant correlation between the effect of monetary reward conditioning on gaze bias and EQ, indicating that the sensitivity to monetary reward might be unrelated to the ability to empathise.

It should be mentioned that we could have compared being mimicked vs neutral faces instead of being anti-mimicked. Both anti-mimicry^8^,^12^,^40^ and no-mimicry^4^,^13^ have been used as comparison conditions in similar previous studies. We chose to use anti-mimicry in order to minimise systematic confounds due to stimulus salience, i.e. a motion-less neutral video might be less salient than a video where a facial expression is performed.

While the sample size of 38–40 included individuals might be relatively small, Bayes factor calculations using a classic reward conditioning effect on gaze bias as prior indicated that it was sufficient to evaluate the evidence for effects studied in both experiments. They further support the presence of a conditioning effect on gaze bias for both experiments. However, Bayes factor was considerably larger for the BeMim experiment. The relatively small Bayes factor for the CARD experiment can be explained by a relatively larger standard error of the before-after conditioning difference in gaze bias (1.6 times bigger as in BeMim), indicating greater inter-subject variability in gaze bias induced by monetary as compared to social rewards.

Future experiments should test general congruence effects on gaze bias in order to establish whether congruence is perceived as more rewarding than incongruence in general or if this effect is specific in the context of mimicry.

In summary, these experiments present a lab-based measure of testing the rewarding nature of mimicry, and demonstrate how trait empathy mediates this relationship. It would be vital to investigate this mimicry-reward relationship in groups associated with low empathy, such as individuals with ASC.

## Methods

### Participants

Forty-six adults (22 male, mean age = 26.59 years, SD = 9.23) without any reported neurological or psychiatric disorders were recruited from the area in and around University of Reading campus and received either a small compensation or credit points for their participation. All participants had normal or corrected-to-normal vision. Ethical approval for the study was obtained from the Research Ethics Committee of the University of Reading and all methods were carried out in accordance with these guidelines regarding all relevant aspects, such as recruitment, information, compensation and debriefing of participants, as well as the nature of the experiments and other collected information. All participants provided informed consent.

### General Procedure

Participants completed the Empathy Quotient^30^ (EQ) online before the lab-based tasks. Every participant took part in both experiments on the same day, and the order of the experiments was counterbalanced across participants. Both the experiments involved a conditioning phase, preceded and followed by a preferential looking phase. In experiment 1, participants were conditioned by repeatedly being mimicked or anti-mimicked by faces (BeMim). In experiment 2, participants were conditioned to associate faces with winning or losing money while playing a card game (CARD). Two non-overlapping sets of 4 faces were used for each task. Participants rated attractiveness and likeability for all faces before and after each conditioning phase. For the preferential looking phase of each task, the 4 faces were presented on a computer screen in randomized order, one pair at a time. Preferential gaze bias was recorded for each face pair before and after the conditioning phase for each experiment. The gaze bias from before the conditioning phase was recorded to serve as a baseline. The instructions for all tasks were presented on the monitor and also read aloud by the experimenter. After completing both experiments, participants completed a questionnaire that evaluated their level of understanding of the purpose of the experiments. They were debriefed afterwards. The whole procedure took 70 to 90 minutes.

## Experiment 1: Effect of being mimicked on gaze bias and rating (**BeMim**)

### Stimuli

Stimuli were derived from the *Amsterdam Dynamic Facial Expression Set (ADFES)* database (http://bit.ly/1dMyC2V). These consisted of 3s videos of 4 different individuals of the same gender as the participant. There were 2 videos per face: one showing a happy and one a sad expression. Each video began with a neutral expression which turned into a happy or sad expression after approximately 1 s and remained until the end of the video. In the preferential looking phase, static images of the same faces with 80% neutral facial expressions were presented in pairs side by side on a black background. These faces were created with Sqirlz Morph 2.1 (http://www.xiberpix.net/SqirlzMorph.html), morphing a neutral face with a happy one. This morphing was done because 100% neutral faces can be perceived as threatening^45^.

### Apparatus

During the conditioning phase and rating measures, stimuli were displayed using E-Prime 2.2 (Psychology Software Tools, PA, USA) on a Viewsonic VE510s monitor (colour TFT active matrix XGA LCD 30.5 cm × 23 cm). For preferential looking, stimuli were presented with TobiiStudio on a Tobii T60 eye tracker monitor (operating at 60 Hz) while participants were seated at a distance of 55 cm to it with their head on a chin rest. The eye tracker was calibrated to the participant’s eyes before each run of the preferential looking phase, using 9 fixation points.

#### EMG Measurement

In order to ensure that participants made the correct facial expression before they saw the face in the video in the BeMim task (see section on Conditioning phase below), facial electromyographic (EMG) responses were recorded during the mimicry conditioning phase, using electrodes placed over the Zygomaticus Major and Corrugator Supercilii. Electrode placement and hardware settings were identical to Sims *et al*., 2012. The timing of the participant’s making the expression was crucial in order to create a subjective feeling that the expression made by the face in the video was in response to the participant’s own expression (e.g. the feeling of being mimicked by the face in case of a congruent trial). The EMG signal was checked manually for each trial by a researcher blind to the experimental conditions. Participants had to achieve a clearly visible signal increase in the correct muscle after the instruction (Zygomaticus Major activity for “happy” and Corrugator Supercilii for “sad”), and before the onset of the facial expression in the video. If this was not the case, the trial was counted as error.

### Procedure

#### Conditioning phase

**Figure 3:**
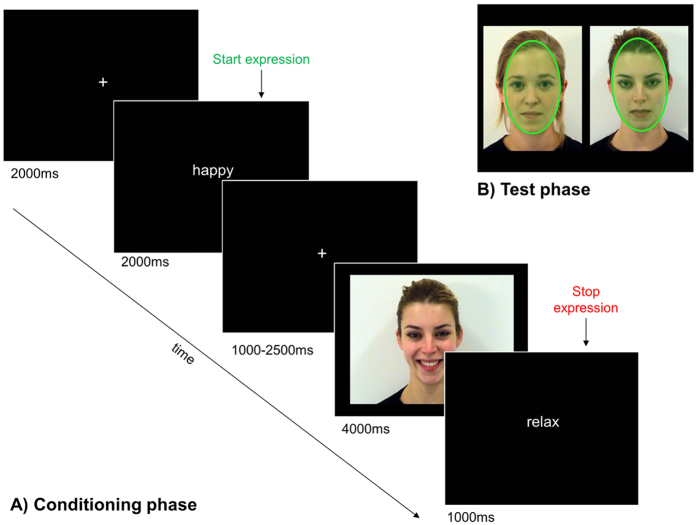
(**A**) BeMim conditioning phase. Participants were first instructed to make an expression and hold it. After a variable delay a video appeared that displayed either the same (mimicking face) or the other expression (non-mimicking face). (**B**) Preferential looking phase. The faces shown previously during the conditioning were presented side by side while recording the participant’s eye gaze behaviour. To ensure their attention to the screen, the participants performed an oddball task where they were asked to press a button when they noticed the fixation cross that was presented during the ISI change its colour. Eye gaze data were extracted for the face region only (elliptic ROI drawn in TobiiStudio) of each face (marked here in green for clarification).

Prior to the experiment, participants read the task instructions and practised their happy and sad expression using a small mirror. After EMG electrode placement, participants completed a short practice session consisting of 8 trials (two for each face, one each for happy and sad expressions) to ensure that the participant was following the instructions and making the correct expressions. Participants were asked to make a happy expression as soon as they saw the word “happy” and a sad expression as soon as they saw the word “sad” on screen, and keep each expression until the word “relax” was displayed. They were instructed to keep a relaxed face when seeing the word “neutral”. Between the expression cue and the word “relax”, a video of a face making a sad or happy expression was displayed for 3 seconds after a 700 ms delay (see Fig. 3A). This expression was either *congruent* or *incongruent* to the participant’s expression or participants kept a neutral face while simply watching the face making a happy or sad expression (*neutral trials*). The 4 faces were associated with 90% (=18 trials; 54 seconds in total) congruent (BeMim90), 90% incongruent (BeNom90), 60% (=12 trials; 36 seconds in total) congruent (BeMim60) and 60% incongruent (BeNom60) trials. All remaining trials (e.g. the remaining 10% trials for the BeMim90 face) were associated with neutral instructions. The BeMim60 and BeNom60 conditions were included in order to prevent participants from easily guessing the underlying conditioning procedure. Out of all participants, only 2 reported to have noticed that one of the faces usually made a congruent and another one an incongruent expression during the conditioning when asked after the experiment. Faces were counterbalanced across participants for these 4 conditions. For each face, half of the trials were associated with a happy expression while the other half was associated with a sad one. There were 20 conditioning trials per face (10 happy, 10 sad), resulting in 80 conditioning trials in total. After 40 trials, participants were given an opportunity to take a break. Each half of the conditioning phase contained the same number of congruent, incongruent and neutral trials as well as the same number of happy and sad video stimuli. Within each half, the stimulus order was randomized.

#### Preferential looking phase

During each preferential looking phase, the participants’ eye tracking data were recorded while they watched the conditioned faces, one pair at a time. Faces were presented in pairs side by side, counterbalanced for the side of the screen (see Fig. 3B), in pseudo-randomized order (using TobiiStudio version 3.1.2.). There were 8 trials per face-pair, presented between 4.4 to 5.3 seconds (jittered to prevent anticipatory looking patterns), followed by a variable inter stimulus interval (ISI) (1.0–1.6 seconds). In order to keep participants focused on the screen they performed an oddball task unrelated to the faces: After 10% of the ISIs, the fixation cross would change its colour to green for 1 second and back to white for 1.0, 1.2, 1.4 or 1.6 seconds. Participants were instructed to click the left mouse button when the fixation cross changed its colour to green and to look wherever they wanted on the screen while the faces were presented. Each run of the preferential looking task (before and after conditioning) took approximately 5 minutes.

### Data analyses

#### Exclusion

Exclusion criteria were defined as follows: (1) Participants whose pupils were not detected by the eye tracker for more than 50% of the total duration of any of the two preferential looking phases. Three participants were excluded on the basis of this criterion. (2) Participants whose gaze to all faces in total was below 10% of the total time in which faces were presented were excluded, which was the case for 5 participants. Overall, 38 participants (17 males) were included in the eye tracking analysis. All but 1 participant (due to missing data) were included in the analysis of the rating data.

#### Normality checks and transformations

The distribution for all variables was tested before analysis, using Shapiro-Wilkinson’s test of normality and log-normality. Parametric and non-parametric tests of statistical inference were used accordingly. In cases where even the distribution of log-transformed variables showed signficant deviation from normality, non-parametric tests and non-transformed variables were used.

#### Eye tracking data analysis

Elliptical regions of interest (ROI) were drawn using TobiiStudio, capturing the face region of each stimulus image (see Fig. 3B). All ROIs had exactly the same size. For each stimulus-face, the *gaze duration* defined as the total time that gaze data was recorded within a face ROI was extracted from TobiiStudio for the BeMim90 vs BeNom90 face pair. From this data, *gaze-bias* was computed as the ratio of gaze duration to mimicking vs non-mimicking face (BeMim90/BeNom90) and then compared between the two preferential looking phases (i.e. before and after conditioning). For correlation analyses, the *gaze-bias-ratio*, defined as gaze bias after conditioning divided by gaze bias before conditioning was calculated.

#### Rating data analysis

Before and after conditioning, participants rated attractiveness and likeability of each face. To test the effect of the conditioning on rating, *Likeability-bias*, *attractiveness-bias*, *Likeability-bias-ratio* and *attractiveness-bias-ratio* were calculated in a similar way as the gaze-bias and gaze-bias-ratio and used for paired-sample tests and correlation analyses, respectively. For all correlation analyses, influence measures (Cook’s D and leverage) were calculated and data points exceeding a cut-off of 4/N were excluded.

As we had strong predictions about the directionality of all effects, 1-tailed statistics were used. All analyses were conducted using SPSS 21 (IBM SPSS Statistics version 21).

## Experiment 2: Effect of learnt reward on gaze bias and rating (**CARD**)

The main purpose of Experiment 2 was to confirm the validity of gaze bias as a metric for learnt reward value by testing whether reward conditioning (using monetary rewards) increases gaze bias for faces conditioned with high vs low rewards.

### Procedure

#### Conditioning phase

The conditioning phase of the CARD experiment closely resembled the one used by Sims *et al*. (2012 and 2014). For a detailed description of the conditioning see Sims *et al*. (2012). In the highest reward (Pos90) condition, participants won 25p in 90% of the trials that were paired with that face. In the lowest reward (Neg90) condition, participants lost 20p in 90% of the trials. Two other conditions Pos60 (participants winning 60% of the trials) and Neg60 (participants losing 60% of the trials) were introduced to prevent participants from guessing the underlying structure of the game. All trials that were neither win nor lose trials were “draw” trials (i.e., neither gain nor loss of money). The faces in the 4 conditions (Pos90, Pos60, Neg60, Neg90) were counterbalanced across participants. The presence of the faces alongside the cards was explained by informing the participants that the faces would play a role in a simple memory task later in the experiment.

#### Preferential looking phase

The preferential looking phase of Experiment 2 was nearly identical to the one of Experiment 1, except for the faces presented. The task, the instructions and the number of trials were identical to the BeMim experiment.

### Data analyses

Exclusion procedure, normality tests and all analyses were conducted in exactly the same way as in the BeMim experiment, using SPSS. Influence measures (Cook’s D and leverage) were calculated for each correlation and data points exceeding a cut-off of 4/N were excluded from correlation analysis.

#### Exclusion

Two participants whose pupil was detected by the eye tracker for less than 50% of the duration of one of the two test phases were excluded. Four further participants were excluded whose gaze duration to all faces in total was below 10% of the total time when faces were presented. Overall, 40 participants (17 males) were included in the eye tracking analysis. All 46 participants were included in the analysis of the rating data.

#### Eye tracking data analysis

*Gaze duration* was extracted for both Pos90 and Neg90 faces (from the condition where they were presented together side by side) and *gaze-bias* to high reward vs low reward face (Pos90/Neg90) was compared between before and after conditioning in a paired sample test. For correlation analyses, the *gaze-bias-ratio* defined as in BeMim was calculated and correlated with EQ.

#### Rating data analysis

To test the effect of the conditioning on rating, *Likeability-bias*, *attractiveness-bias*, *Likeability-bias-ratio* and *attractiveness-bias-ratio* were calculated in the same way as in the BeMim experiment and used for paired sample tests and correlation analyses.

#### External Validity check

To further validate the gaze bias metric in addition to reports from the literature, it was tested for a correlation with likeability-bias-ratio.

#### Effect of awareness regarding the manipulation

Unlike in the BeMim experiment where only two participants could figure out the nature of the manipulation, approximately half of the participants were able to name the manipulation of the CARD experiment (that they won with certain faces and lost with others) within the questionnaire completed after the study. Therefore gaze-bias-ratio, attractiveness-bias-ratio and likeability-bias-ratio were compared between those participants who detected the manipulation and those who didn’t (using an independent samples test) to investigate the dependency of the conditioning effect on this knowledge.

## Additional Information

**How to cite this article**: Neufeld, J. and Chakrabarti, B. Empathy Modulates the Rewarding Effect of Mimicry. *Sci. Rep.* **6**, 27751; doi: 10.1038/srep27751 (2016).
